# The Procreation of Defectives

**Published:** 1908-02

**Authors:** A. A. Merrill

**Affiliations:** Boston


### The Procreation of Defectives.

BOSTON, April 15, 1907.

*Mr. Editor:*

Your editorial on the "Procreation of Defectives" in the issue of March 28, suggests to me a thought which I would like to put before your readers.

The bill before the Legislature of Indiana mentions heredity as playing a most important part in the transmission of crime, idiocy and imbecility and suggests that upon the approval of a board of surgeons, a surgical operation shall be performed upon those considered to be confirmed criminals, idiots, rapists and imbeciles. In commenting on this, you state that in the present condition of knowledge such legislation would do little to help matters and might lead to serious abuses.

It is true that we can not be sure that the child will inherit the tendencies of the parent, but the probability is that the child of the criminal, the imbecile, or the degenerate, will be born handicapped for life, both physically and mentally.

It is also true that the question of race improvement does not rest entirely on heredity. Nevertheless, we must remember that not only do the parents furnish this factor to their children, but the condition of the parents fixes the environment of the most important periods of life, the formative periods of infancy and youth. Certainly no criminal, no insane person, and no degenerate, can furnish a proper environment for the child, whatever tendency he may transmit.

The method of checking procreation mentioned in the bill from which you quote is a surgical operation, but no such thing is necessary. We now know that X-ray treatment will produce sterility in the male. This treatment is painless, it is easy to give and as it does not produce impotence, there would seem to be no objectionable features connected with it. The substitution of this treatment for a surgical operation would, it seems to me, put the matter in a different light.—*A. A. Merrill, Boston Medical and Surgical Journal.*

---

NIGHT-TERRORS IN CHILDREN.—

℞ Potassii bromidi . . . . . . . . . . . . . . . . . . . . . . grm. 0.5
Tinct. hyoscyami. . . . . . . . . . . . . . . . . . . . . . . . gtt. 10
Syr. simp. . . . . . . . . . . . . . . . . . . . . . . . . . . . grm. 15
Aq. . . . . . . . . . . . . . . . . . . . . . . . . . . . . . . . grm. 10

M. Sig. To be taken in a single dose on going to bed.—*Jour. de med. de Paris.*

